# Knowledge and attitude towards preconception care and associated factors among women of reproductive age with chronic disease in Amhara region referral hospitals, Ethiopia, 2022

**DOI:** 10.1186/s12905-024-02994-4

**Published:** 2024-03-19

**Authors:** Muluken Demeke, Fisseha Yetwale, Zerfu Mulaw, Daniel Yehualashet, Anteneh Gashaw, Berihun Agegn Mengistie

**Affiliations:** 1 Department of Midwifery, College of Medicine & Health Sciences, Dilla University, Dilla, Ethiopia; 2 School of Midwifery, College of Medicine & Health Sciences, University of Gondar, Gondar, Ethiopia

**Keywords:** Preconception care, Knowledge, Attitude, Chronic disease, Ethiopia

## Abstract

**Introduction:**

Preconception care (PCC) is an important window to target maternal morbidity and mortality, especially for women with chronic diseases. However, little is known about knowledge and attitudes towards preconception care among women with chronic disease. Therefore, this study aimed to assess knowledge and attitude towards preconception care and associated factors among women of reproductive age with chronic disease in Amhara region referral hospitals, Ethiopia, 2022.

**Method:**

A multicenter cross-sectional study was conducted in Amhara region referral hospitals from April 15 to June 1, 2022. A total 828 women of reproductive age with chronic disease in four referral hospitals were selected using a stratified and systematic random sampling technique. Data was collected by using a structured interviewer-administered questionnaire and chart review. Bivariate and multivariable logistic regression analyses were carried out. An Adjusted Odds Ratio (AOR) with 95% Confidence Interval (CI) was computed to see the strength of association between outcome and independent variables. P-value < 0.05 was considered as statistically significant.

**Results:**

This study found that 55.6% of respondents had a good knowledge of preconception care, and 50.2% had a good attitude towards PCC. Formal education (AOR: 1.997, 95% CI: 1.247, 3.196), primiparity (AOR: 2.589, 95% CI: 1.132, 5.921), preconception counseling (AOR: 3.404, 95% CI: 2.170, 5.340), duration of disease ≥ 5 years (AOR: 6.495, 95% CI: 4.091, 10.310) were significantly associated with knowledge of PCC. Older age (≥ 35years) (AOR: 2.143, 95% CI: 1.058, 4.339), secondary education and above (AOR: 2.427, 95% CI: 1.421, 4.146), history of modern family planning use (AOR: 2.853 95% CI: 1.866, 4.362), preconception counseling (AOR: 2.209, 95% CI: 1.429, 3.414) and good knowledge of PCC (AOR: 20.629, 95% CI: 12.425, 34.249) were significantly associated with attitude towards PCC.

**Conclusions:**

Women’s knowledge and attitude towards preconception care were found to be low. Important measures include promoting secondary education and carrying out awareness campaigns, incorporating preconception counseling into routine medical follow-up care, and encouraging the use of modern family planning methods.

## Background

Preconception care is the provision of biomedical, behavioral, and social health interventions to women and couples before conception occurs [1]. The overarching goal is to improve maternal and child health in the short and long term by reducing behaviors that contribute to unfavorable maternal and child health outcomes [1, 2].

According to the World Health Organization (WHO), 295,000 women die worldwide each year due to complications related to pregnancy or childbirth. Every day, about 810 women die from pregnancy- or childbirth-related complications, with 94% of all maternal deaths occur in low- and ,middle-income countries (LMICs) [3]. In Ethiopia, as reported in the 2016 Ethiopian Demographic Health Survey (EDHS), maternal mortality was 412 per 100,000 live births, and neonatal mortality was 29 per 1,000 live births [4]. Most of these complications occurring during pregnancy, exist prior to pregnancy, and worsen during pregnancy, particularly if not managed as part of preconception care [3].

In LMICs, preconception care is often low or nonexistent, and even where it exists, it does not adequately support women to enter pregnancy with optimal health [5]. Ethiopia, like other African countries, is struggling to reduce maternal and neonatal mortality and morbidity, but medical disorders impose an extra burden on the health care system, and maternal and neonatal mortality remain high [6].

Chronic diseases are on the rise in both developed and developing countries and have more than doubled among reproductive-age women in many African countries, including Ethiopia [7]. Ethiopia is experiencing a rise in the prevalence of chronic diseases, with the Amhara region exhibiting among the highest prevalence [8, 9]. Globally, half of pregnancies are unintended, and in Ethiopia, one-third of pregnancies are unintended [4, 10]. Moreover, chronic diseases such as diabetes mellitus, hypertension, thyroid disease, epilepsy, and renal disease during pregnancy lead to a multitude of maternal and fetal consequences, including preterm birth, intrauterine growth retardation, fetal loss, congenital malformations, preterm delivery, and perinatal mortality of the offspring [11, 12]. Nevertheless, preconception care for women with chronic illnesses receives minimal attention.

In Ethiopia, the average time for the first antenatal care (ANC) visit is five months [13]. This is considered too late to address risk factors [14]. Therefore, preventive intervention is invaluable [15]. Women who are knowledgeable about preconception care can optimize their health before getting pregnant and engage in health-seeking behaviors [16].

Preconception care aims to enhance women’s knowledge and attitudes towards preconception health care [17]. However, lack of knowledge about preconception care has been recognized as one of the biggest hurdles to its application, and is one of the key reasons that prevent couples from getting it [18, 19]. Poor maternal health-seeking attitudes can lead to undesirable consequences, including low birth weight and premature birth [20].

Although women with chronic diseases tend to develop complications, many studies in our country as well as in the study area have focused on knowledge of preconceptional care among reproductive-age women without medical disorders, despite the fact that preconception care is absolutely indispensable for these women [21]. Studies are scares about knowledge and attitudes towards preconception care among reproductive-age women with chronic disease in Ethiopia. Successful interventions require not only the effectiveness of the intervention but also an understanding of the knowledge, attitude, and behavior of the target population [11]. This study, therefore, aimed at assessing knowledge and attitude towards preconception care and associated factors among women of reproductive age with chronic disease in Amhara region referral hospitals, Ethiopia, 2022. The implication of this study is to develop targeted strategies and tailor preconception health care service for women with chronic disease.

## Methods

### Study design, area and period

A multicenter cross-sectional study was conducted in Amhara region referral hospitals from April 15 to June 1, 2022. The Amhara region is Ethiopia’s second-largest region, located in the country’s north, and has 11 administrative zones. The population of the Amhara region was estimated to be 28 million in mid-2018 [22]. There are 80 hospitals, 220 health centers, and 2941 health posts in the Amhara region. In this region, there are eight referral hospitals, namely Gondar University, Felege-Hiwot, Tibebe-Giyon, Woldia, Dessie, Debre-Markos, Debre-Tabor, and Debre-Birhan Comprehensive Specialized Hospitals. The study was carried out in four randomly selected referral hospitals: Namely, Debre-Tabor, Felege-Hiwot, Dessie, and Debre-Marikos Comprehensive Specialized Hospitals. Each hospital has two medical follow-up Outpatient Department/clinics (OPD), which cater to the population with medical disorders. As per the information collected from hospitals (nurse and log book), the previous 6-week report indicates that 1700 reproductive-age women with chronic diseases came for follow-up. All women of reproductive age who were diagnosed with chronic diseases and available during data collection were included in this study. Chronic diseases included Diabetes mellitus, Hypertension, Cardiac disease, Thyroid disease, Epilepsy, Asthma, Autoimmune disease, Stroke, Renal disease, and Hepatic Disease.

### Sample size and sampling procedure

Sample size was calculated using the single proportion formula with the following assumptions:

1 $$n = {({\rm{Za}}/2)^2}\,{\rm{P}}\,(1 - {\rm{P}})/{{\rm{d}}^2}$$

Where:

- *n* is the minimum sample size needed.
- D is desired precision (5%).
- P is assumed to be 50% since no similar study had been conducted on reproductive-age women with chronic diseases.
- Z _a/2_ is 1.96 at a confidence level of 95%.

Based on these assumptions, the sample size was 384. By adding a 10% non-response rate, the final sample size became 422. Since the sampling technique was multistage, it was multiplied by two, resulting in a final sample size of 844 [23].

A multistage stratified sampling procedure was employed. The Amhara region has eight referral hospitals. The study population was stratified into these eight referral hospitals, and among them, four referral hospitals were randomly selected by lottery method. Then the sample size was allocated proportionally to each randomly selected referral hospital based on the number of reproductive-age women with chronic disease seen in the follow-up OPD over six weeks, determining the skipping interval. Afterward, a systematic random sampling technique was employed, with the first participant chosen randomly, followed by selecting every 2nd interval.

### Operational definition

Knowledge: Women’s knowledge of preconception care was measured using 32 preconception care knowledge questions and scored out of a total of 32 points. The mean was utilized as the cutoff point, women’s knowledge was divided into two categories [24, 25].

#### Good knowledge

respondents who scored greater or equal to the mean were categorized as having good knowledge of preconception care.

#### Poor knowledge

respondents who scored less than the mean were categorized as having poor knowledge of preconception care.

#### Attitude

women`s attitude towards preconception care was measured using six questions. Each question has 5-point Likert scale of “1”, “2”, “3”, “4”, and “5”, denoting strongly disagree, disagree, neutral, agree, and strongly agree, respectively. With the mean as a cutoff point, women’s attitude was divided into two categories [24].

#### Good attitude

respondents who scored greater or equal to the mean to preconception care attitude questions were categorized as having a good attitude towards preconception care.

#### Poor attitude

respondents who scored less than the mean to preconception care attitude questions were categorized as having a poor attitude towards preconception care.

**Good adherence to follow-up appointment**; women who attended 70% and above of the appointments (seven out of the last ten appointments) [26].

#### Comorbidity

having more than one chronic disease in a woman at the same time [27].

### Data collection tools and procedures

Data were collected using an interviewer-administered, pre-tested, and structured questionnaire, as well as chart review to ascertain the diagnosis and comorbidity. The questionnaire was adapted by reviewing different literature and contextualized to the situation [24, 28–32]. The questionnaire was checked with a Cronbach alpha of 0.90 for knowledge-assessing tools and 0.87 for attitude-assessing tools. The questionnaire includes sections on socio-demographic factors, obstetric, family planning, and disease-related factors, as well as sections for knowledge and attitude questions. Data were collected by four BSc nurses who work at each referral hospital.

### Data quality control

To keep the quality of data, the questionnaire (English version) was translated into Amharic and then translated back to English by two different persons: the forward translation by the principal investigator and the back translation by another clinical midwifery student of University of Gonder to ensure consistency and accuracy. The content’s validity was assessed by three assistant professors of clinical midwives and one gynecologist. Two weeks before data collection, the questionnaire was pre-tested at Woldia Comprehensive and Specialized Hospital on 10% of the final sample by the principal investigator; it was not part of data collection site. After pre-testing, necessary adjustments were made accordingly. Data were collected by four BSc nurses who work at each of four referral hospitals. Both data collectors and supervisors were given one-day training before the actual work, including the aim of the study, procedures, and the way to collect data, as well as maintaining the confidentiality of the information gained from the respondents. Supervision throughout the data collection was carried out.

### Data processing & analysis

After data collection, each questionnaire was manually checked for completeness. Then, the data was coded, entered using Epidata V4.6.0.2, and exported to SPSS for data checking, cleaning, and logistic regression. Frequencies were used to check for missing observations. Descriptive statistical analysis including frequencies, mean, and standard deviation for continuous variables and percentages for categorical variables was conducted. A Pearson’s chi-squared test was performed to examine the association between individual-level factors and the outcome variable. Finally, bi-variate and multivariable logistic regression analysis was carried out to check the significant association between dependent and independent variables, with statistical significance considered at *P* < 0.05 and AOR with a 95% confidence interval.

## Results

### Socio-demographic characteristics

From a total of 844 study participants required for the study, 828 reproductive-age women with chronic disease participated, giving a total response rate of 98.1%. The mean age of the women was 33.2 years, with a standard deviation of ± 8.4 years. More than three-fourth of respondents, 674 (81.4%), were followers of Orthodox Christianity, followed by Islam, which accounts for 139 (16.8%). Concerning the educational status of respondents, more than three-fourths of participants 699 (84.4%), attended formal education. The majority of respondents, 745(90%), were married, and most of the respondents, 571 (69%), were housewives. More than half of the respondents 433 (52.3. %) were living in urban areas. Regarding the participant’s husband’s education, 290 (38.9%) attended primary education, and half of the participant’s husband’s occupation, 379 (50.9%), was in private business (See Table 1).

**Table 1:** Socio-demographic characteristics of reproductive-age women with chronic disease in Amhara region referral hospitals, northern Ethiopia, 2022 (*N* = 828)

Variables	Frequency (N)	Percent (%)
**Age**		
15–24	164	19.8
25–34	299	36.1
35–49	365	44.1
**Religion**		
Orthodox	674	81.4
Muslim	139	16.8
Protestant	15	1.8
**Educational status**		
No formal education	221	26.7
Primary education	307	37.1
Secondary education	218	26.3
College and above	82	9.9
**Occupation**		
House wife	571	69
Private business	96	11.6
Government employ	41	5
Student	74	8.9
Daily labor	11	1.3
Farmer	35	4.2
**Marital status**		
Married	745	90
Single	68	8.2
Divorced	7	0.8
Widowed	8	1
**Husband educational status (N = 745)**		
No formal education	155	20.8
Primary education	290	38.9
Secondary education	201	27
College and above	99	13.3
**Husband Occupation (N = 745)**		
Private business	379	50.9
Government employ	118	15.8
Student	12	1.6
Daily labor	37	5
Farmer	199	26.7
**Monthly income**		
< 1000ETB(Ethiopian Birr)	78	9.4
1000-1999ETB	442	53.4
> 2000ETB	308	37.2
**Residence**		
Rural	395	47.7
Urban	433	52.3
**Having Mass Media to access health related information (TV/radio)**		
Yes	567	68.5
No	261	31.5
**Having mobile phone to access health related information**		
Yes	549	66.3%
No	279	33.7%

### Obstetric characteristics of respondents

Among all respondents, 722 (87.2%) were previously pregnant; of them, 218 (26.3%) were primiparous, and 491 (59.2%) were multiparous.

The majority of respondents, 601(83.2%) had at least one ANC follow-up, 591(81.9%) of respondents had a history of institutional delivery, and 234(32.4%) of respondents had a history of postnatal care for their recent pregnancy. Nearly one-third, 213 (29.5%) of respondents had a history of adverse birth outcomes. Among them, abortion accounts for 94 (43.2%), stillbirth 39 (18.3%), congenital anomaly 24 (11.3%), LBW (low birth weight) 19 (8.9%), preterm birth 17 (8%), and neonatal death 31 (14.6%). Four hundred forty-three (61.4%) of respondents had planned pregnancy history for their recent pregnancy. Most of the respondents, 488 (58.9%), had a history of modern family planning; among them, 255 (52.2%) participants used injectables, 113 (22.9%) used oral contraceptives, 86 (17.6%) used implants, 20 (4.09%) used IUCD (intra-uterine contraceptive device), and 15 (3.07%) used others (condom, post-pills).

### Preconception care information

**Fig. 1:**
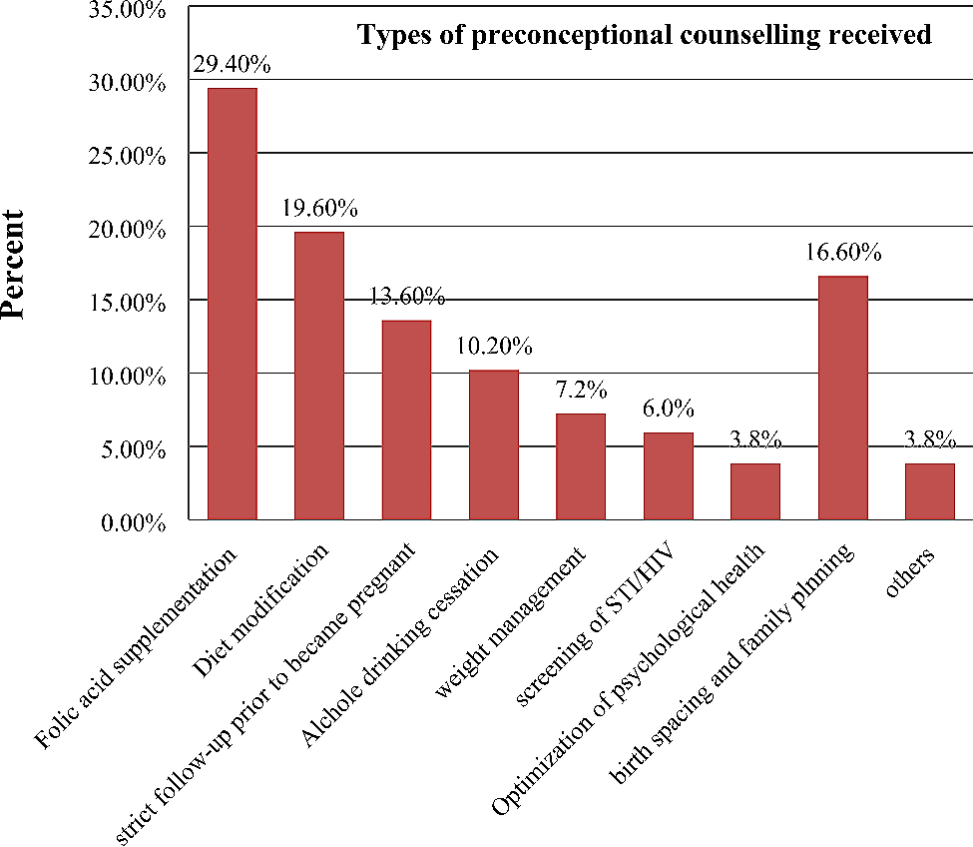
Type of counseling received on preconception care among women of reproductive age with chronic disease in Amhara region referral hospitals, northern Ethiopia, 2022

Less than one-third of respondents, 237 (28.6%), received pre-conception advice from healthcare providers; among them, 69 (29.4%) participants received counseling about folic acid supplementation, 46 (19.6%) participants were counseled about diet modification, and 32 (13.6%) participants were counseled about strict follow-up prior to becoming pregnant (multiple response questions). (See Fig. 1)

### Respondents’ chronic illness profile

A predominant proportion of respondents, comprising more than half, reported having diabetes mellitus (27.2%) and hypertension (25.7%), with 225 and 213 individuals, respectively. Among all respondents, 64 (7.7%) had cardiac disease, 87 (10.5%) had thyroid disease, 64 (7.7%) had epilepsy, 47 (5.7%) had asthma, 78 (9.4%) had renal disease, and 50 (6.1%) reported other disorders.

The median month of disease duration since diagnosis was 48 months, with an IQR (interquartile range) of 29 months. The minimum duration was one month, and the maximum was 180 months. Among the respondents, 754 (91.1%) had ten or more follow-ups, and within this group, 648 (87%) exhibited good adherence to follow-up appointments. Additionally, 164 (19.8%) had co-morbid diseases.

### Women’s knowledge of preconception care

Out of the total respondents, 460 (55.6%) had good knowledge of preconception care with a 95% CI (confidence interval) ranging from 52.3 to 59.1%.

Among the total of 828 respondents, 503 (60.7%) have ever heard of preconception care. Health professionals were the major source of information for 237 (47.3%) respondents, followed by mass media 134 (26.8%), internet 97 (19.4%), friends/relatives 80 (16%), and school 18 (3.6%).

Most of the respondents, 462 (55.8%), responded that preconception care is needed for both men and women, while 192 (23.2%) responded that preconception care is needed for women only. Concerning the site of preconception care, 401 (48.4%) of respondents said that health institutions are the site of preconception care, and 361 (43.6%) said that both homes and health institutions are sites of preconception care.

### Women’s knowledge of preconception health issues

The findings of the study on women’s knowledge of preconception health issues revealed that 718 (86.7%) of participants responded that medical checkups are necessary prior to pregnancy, and 550 (66.4%) responded that preparation for pregnancy through preconception care is best before getting pregnant (see Table 2).

**Table 2:** Women’ Knowledge of preconception health issues in Amhara region referral hospitals, northern Ethiopia, 2022

Variable (*N* = 828)	Options	Frequency(N)	Percentage (%)
PCC is care given to all women before pregnancy to make them healthier	Yes	527	63.6
	No	301	36.3
PCC is not the same as antenatal care	Yes	450	54.3
	No	378	45.6
PCC enables healthier babies to be born	Yes	481	58.1
	No	347	41.9
Preparation for pregnancy through PCC is best before getting pregnant	Yes	550	66.4
	No	278	33,6
Preconception care ensures diseases are under control before pregnancy	Yes	520	62.8
	No	308	37,2
PCC prevents unintended pregnancies and promotes optimal birth spacing	Yes	362	43.7
	No	466	56.3
Is it necessary for the health personnel to have a say (advise) as to when you can get pregnant?	Yes	400	48.3
	No	428	51.7
Is it important to have a medical check-up before you go on to get pregnant?	Yes	718	86.7
	No	110	13.3

### Women’s knowledge of untreated health problems and behaviors affecting maternal health and pregnancy outcome

Of all respondents, 624 (75.4%) noted cigarette smoking and 620 (74.9%) noted alcohol consumption affecting maternal health and pregnancy outcomes. Regarding women’s knowledge of untreated health conditions affecting maternal health and pregnancy outcome, the most frequently mentioned items were cardiovascular illnesses 602 (72.7%), HIV 526 (63.5%) and diabetes mellitus 430 (51.9%) (see Table 3).

**Table 3:** Women’s knowledge of untreated health problem, and behaviors affecting the maternal health and pregnancy outcome in Amhara region referral hospitals, northern Ethiopia, 2022

Variable (*N* = 828)		Frequency(N)	Percent (%)
Diabetes mellitus	Yes	430	51.9
	No	398	48.1
Epilepsy	Yes	250	30.2
	No	578	69.8
Obesity	Yes	266	32
	No	562	67.9
STIs and HIV/AIDS	Yes	526	63.5
	No	302	36.5
Cardiovascular disease	Yes	602	72.7
	No	226	27.3
Stress and depression	Yes	420	50.7
	No	408	49.3
Genetic disease	Yes	348	42
	No	480	58
Cigarette smoking	Yes	624	75.4
	No	204	24.6
Alcohol consumption	Yes	620	74.9
	No	208	25.1
Exposure to environmental hazard	Yes	532	64.3
	No	144	35.7
Un-prescribed drug intake	Yes	636	75.6
	No	202	24.4
Gender based violence	Yes	464	56.0
	No	364	44

### Women’s knowledge of what should be done before pregnancy

The most frequently mentioned items in women’s knowledge of things that should be done before pregnancy were as follows: 746 (90.1%) respondents stated that pregnancy should be planned, 752 (90.8%) avoid cigarette smoking and 750 (90.6%) avoid un-prescribed medicines. Less frequent mentioned items were taking folic acid 234 (28.3%), tetanus vaccine 248 (30.0%), and maintaining weight 366 (44.2%) (see Table 4).

**Table 4:** Women’s Knowledge of component of preconception care in Amhara region referral hospitals, northern Ethiopia, 2022

Variables (*N* = 828)		Frequency (N)	Percent (%)
Pregnancy should be planned	Yes	746	90.1
	No	2	0.2
	Don’t know	80	9.7
Taking folic acid	Yes	234	28.3
	No	18	2.2
	Don’t know	576	69.6
Weight should be maintained	Yes	366	44.2
	No	12	1.4
	Don’t know	450	54.3
Diet should be modified	Yes	380	45.9
	No	12	1.4
	Don’t know	436	52.7
Regular exercise	Yes	502	60.6
	No	6	0.7
	Don’t know	320	38.6
Substance should be avoided before pregnancy	Yes	664	80.2
	No		
	Don’t know	164	19.8
Cigarette smoking should be avoided	Yes	752	90.8
	No	0	0
	Don’t know	76	9.2
Alcohol consumption should be avoided before pregnancy	Yes	718	86.7
	No	40	4.8
	Don’t know	70	8.5
illicit drugs should be avoided before pregnancy	Yes	750	90.6
	No	16	1.9
	Don’t know	62	7.5
Healthy environment should be created before pregnancy	Yes	474	57.2
	No	8	1
	Don’t know	346	41.8
Free from stressors	Yes	522	63.0
	No	8	1.0
	Don’t know	298	36.0
TT vaccination	Yes	248	30.0
	No	14	1.7
	Don’t know	62	7.5

### Predictors of knowledge of preconception care

Bi-variable analysis showed that factors such as age, formal education, occupation of respondents, residence, mass media, primiparity, history of modern family planning, preconception counseling, and duration of disease had a P value ≤ 0.25, and were taken into the final model.

In the multivariable logistic regression, four factors were found to have an independent association with the knowledge of PCC. Women who attended primary education and above were nearly two times more likely to have good knowledge of PCC compared to those with no formal education (AOR: 1.997; 95%CI: 1.247, 3.196).

Primiparous women were 2.5 times more likely to possess good knowledge of PCC compared to nulliparous women (AOR: 2.589; 95%CI: 1.132, 5.921). Women who had received preconceptional counseling were 3.4 times more likely to have good knowledge of PCC compared to their counterparts (AOR: 3.404; 95%CI: 2.170, 5.340). Women whose duration of disease was more than five years were 6.4 times more likely to have good knowledge of PCC compared to their counterparts (AOR: 6.495; 95%CI: 4.091, 10.310) (see Table 5).

**Table 5:** Factors associated with knowledge of preconception care among women of reproductive age with chronic disease in Amhara region referral hospitals, Northern Ethiopia, 2022

Variables (*N* = 828)	Knowledge			COR (95% CI)	AOR( 95% CI)
	Good		Poor		
Educational status					
No formal education	65		156	1	1
Primary education	141		166	2.039(1.413, 2.940) *	1.997(1.247,3.196)**
Secondary education and above	254		46	13.252(8.646, 20.312) **	14.775(8.153, 26.778)***
Parity					
Nullipara	64	55		1	1
Primipara	157	61		2.211 (1.296, 3.339)*	2.589(1.132,5.921) *
Multipara	239	252		0.815(0.530, 1.219)	1.009(0.429,2.375)
PCC Counseling received					
Yes	186	51		4.219 (2.975, 5.984)**	3.404 (2.170,5.340)***
No	274	317		1	1
Duration of disease					
< 5 years	262	306		1	1
>=5years	198	62		3.730 (2.683, 5.186)**	6.495(4.091, 10.310)***

*Note* 1 reference category

AOR: adjusted odd ratio

COR: crude odd ratio

* Shows p value < 0.05

**-p value < 0.01

***-p value < 0.001

### Women’s attitude towards preconception care

Out of the total respondents, 416 (50.2%) with (95% CI: 46.9; 53.5) respondents exhibited a good attitude towards preconception care.

With respect to individual attitude items, 329 (39.7%) respondents agreed and 181 (21.9%) strongly agreed that a hospital setting is the best place to provide preconception care. Of all respondents, 345 (41.7%) agreed and 156 (18.8%) strongly agreed that preconception care is a high priority for women with major medical illnesses (see Table 6).

**Table 6:** Attitude towards preconception care among women of reproductive age with chronic disease in Amhara region referral hospitals, northern Ethiopia, 2022

Parameters N(828)	Strongly disagree		Disagree		Neutral		Agree		Strongly agree	
	N	%	N		%		N			
Hospital or clinic is the best place to provide PCC	6	0.7	16	1.9	296	35.7	329	39.7	181	21.9
PCC has any positive effect on pregnancy outcome	12	1.4	108	13.0	322	38.9	289	34.9	97	11.7
PCC can improve women’s health	10	1.2	142	17.1	280	33.8	295	35.6	101	12.2
PCC is an important health issue for women of childbearing age	6	0.7	108	13.0	290	35.0	303	36.6	121	14.6
PCC is a high priority for women with major medical illness to plan pregnancy	10	1.2	58	7.0	259	31.3	345	41.7	156	18.8
I am the most suitable person plan to get PCC	14	1.7	64	7.7	293	35.4	289	34.9	168	20.3

### Predictors of attitude towards preconception care

Results of the bi-variable analysis showed that age, formal education, occupation, residence, mass media, primiparity, history of modern family planning use, preconception counseling, duration of disease, comorbidity, and knowledge of PCC had a P value of ≤ 0.25 and were included in the final model.

In the multivariable logistic regression analysis, five factors were identified to have a statistically significant association with attitudes towards preconception Care. Women aged 35–49 were 2.1 times more likely to have a good attitude towards PCC compared to women aged 15–24 (AOR: 2.143; 95%CI: 1.058, 4.339). Women who attended secondary education and above were 2.4 times more likely to have a good attitude toward PCC compared to those with no formal education (AOR: 2.427; 95% CI: 1.421, 4.146). Women who had a history of modern family planning use were 2.8 times more likely to have a good attitude towards preconception care compared to their counterparts (AOR: 2.853; 95%CI: 1.866, 4.362). Women who had received preconceptional counseling were 2.2 times more likely to have a good attitude towards PCC compared to their counterparts (AOR: 2.209; 95%CI: 1.429, 3.414). Women with good knowledge of PCC were 20.6 times more likely to have a good attitude towards PCC compared to their counterparts (AOR: 20.629; 95%CI: 12.425, 34.249) (see Table 7).

**Table 7:** Factors associated with attitude towards preconception care among women of reproductive age with chronic disease in Amhara region referral hospitals, northern Ethiopia, 2022

Variables (*N* = 828)	Attitude		COR (95% CI)	AOR (95% CI)
	Good	Poor		
**Age**				
15–24	75	89	1	1
25–34	173	126	1.629 (1.110, 2.391)*	1.604,(0.892, 2, 886)
35–49	168	197	1.012(0.699, 1.465)	2.143(1.058, 4.339)*
**Educational status**				
No formal education	60	161	1	1
Primary education	166	141	3.159 (2.178, 4.582)**	0.688 (0.375, 1.264)
Secondary education and above	190	110	4.635 (3.175, 6.765)**	2.427 (1.421,4.146) **
**History of modern family planning use**				
Yes	315	173	4.309(3.200, 5.802 )**	2.853 (1.866,4.362)***
No	100	239	1	1
**PCC Counseling received**				
Yes	180	57	4.750 (3.380, 6.676)**	2.209 (1.429, 3.414)**
No	236	355	1	1
**Knowledge**				
Good	356	104	17.572(12.352, 24.997)**	20.629(12.425, 34.249)***
Poor	60	308	1	1

*Note*: 1-reference category

AOR: adjusted odd ratio

COR: crude odd ratio

*- p value < 0.05

** - p value < 0.01

***- p value < 0.001

## Discussion

In this study, the participants’ knowledge of preconception care was found to be 55.6% (95% CI: 52.3; 59.1). This finding is in line with the study done in Jinka town (55.2%) [25], but it is higher than the study done in Hawassa, Ethiopia (20% ) [33]. This variation may be attributable to differences in participants’ level of information, as evidenced by a higher percentage of participants (60.7%) in the current study who had heard about preconception care, while in the Hawassa study, only 34% were aware of it. Similarly, this study also higher than studies conducted in West Shewa, Ethiopia (26.8%) [34], Mana district, southwest Ethiopia (21.3%) [35], and Adet, northwest Ethiopia (27.5%) [29]. This variations is likely due to differences in study settings: the current study was conducted in a health institution, while the studies in West Shewa, Mana district, and Adet were community-based; women who had contact with health care providers may have received more information about preconception care, and on top of that women with chronic diseases may have paid more attention to their health before getting pregnant compared to others.

Likewise, it is higher than a study done in Malaysia (48.6%) [30]. This is due to difference in the composition of the study’s population. In the current study, participants were women with chronic diseases attending the follow-up clinic, potentially being exposed to preconception information during routine follow-ups. In contrast, the participants in Malaysian study were high-risk pregnant women at the time of their first ANC booking.

The finding of this study is higher than studies done in Saudi Arabia (22.8%) [36], Nepal (7%) [37], and Ghana (23.5%) [28]. The variations could be attributed to differences in measuring the level of knowledge, and sampling technique used. In the current study, knowledge levels were categorized into two groups, whereas the study in Saudi Arabia categorized them into five. Notably, the Nepal and Ghana studies employed non-probability sampling techniques to select participants, potentially compromising the results.

The finding of this study is lower than a study done in Addis Abeba, Ethiopia (68.6%) [38]. This variation may be due to differences in residence, and educational background of respondents. In the current study, approximately half (52%) of the respondents were living in urban areas, whereas in the Addis Ababa study, the majority of respondents (84.6%) were urban residents. Additionally, in the current study, more than half (63.8%) of the respondents attended primary education and lower, while in the Addis Ababa study, more than half (59.5%) had attended secondary education or higher. Variations in residency and educational status can have a consequence on information access, healthcare facilities, and socioeconomic issues.

On the other hand, the finding of this study is lower than studies done in Osun State, Nigeria (65.3%) [39], Ibadan, Nigeria (59.9%) [24], and Iran (68.8%) [40]. This difference may be due variation in availability and accessibility of preconception healthcare services, socioeconomic differences, and media coverage.

The result of this study showed primary education was significantly associated with good knowledge of preconception care. This finding is consistent with a study done in Adet [29]. Moreover, secondary education was significantly associated with good knowledge of preconception care. This finding is supported by studies done in Jinka [25], Hawassa [33], west shewa [34], Adet [29], and Nigeria [24]. This is due to the fact that as the level of education increases, critical thinking also increases; in addition, media exposure increases and this makes them to access health information easily from different sources like internet [41].

This study found that being primiparity was a significant predictor of good knowledge of preconception care. This is because women are exposed to preconception care information at ANC follow-up, during institutional delivery, or during postnatal care.

Moreover, this study revealed that preconception counseling was significantly associated with good knowledge of preconception care. The finding of this study is supported by studies done in Addis Abeba, Ethiopia [38] and Egypt [42]. This is due to the fact that preconception counseling is a valuable source of information about preconceptional care. Regular interactions and follow-ups visit associated to counseling contribute a deeper understanding of PCC [43].

Furthermore, this study demonstrated that a longer duration of disease (lasting five years and above) was significantly associated with good knowledge of preconception care. This could be due to extended follow-up years, fear, experience of complications, and increased awareness of risks; as a result, women may seek pre-conceptional information from various sources over time.

In this study, the participants’ attitude towards preconception care was found to be 50.2% (95% CI: 46.9; 53.5). This finding is in line with a study done in India (52%) [44]. However, it is higher than a study done in Mizan-Aman, Ethiopia (33.7%) [16]. This could be the difference in the study settings. In the current study, participants were women who presented in a follow-up clinic, whereas in Mizan Aman, participants were in the community. Women who had contact with healthcare providers in the current study may have received information about preconception care, contributing to a more positive attitude towards it. Similarly, the finding of this study is higher than studies done in Mashhad Iran (20.9%) [45], and Ghana (20%) [28]. This could be due to differences in measuring the level of attitude and sampling tequnique used. In the current study, attitude were classified as good and poor while in the Iran study, attitude were classified as weak, neutral, and good. The use of this rating system in Iran might have resulted in a lower percentage of attitudes. Additionally, in the current study, the probability sampling technique was used, which enhances representativeness of study sample. In contrast, the Ghana study used non probability sampling technique which lacks representativeness, potentially affecting the result.

On the other hand, this study is lower than study done in Iran (98.9%) [46], Eswatini (75.4%) [47], Kelantan (98.5%) [31], Nigeria Ibadan (53.9%) [24], and Sudan (83%) [48]. This variation may be due to differences in socio-economic status and the availability and accessibility of preconception care.

This study showed that older age (35–49 year) was significantly associated with a good attitude towards preconception. This finding is consistent with a study done in Iran [40]. Older women may acquire information during ANC, family planning, delivery. Additionally, experience from previous pregnancies could play a role in shaping their attitudes and motivate them to have a more positive outlook towards preconception care.

The finding of this study revealed that level of education was significantly associated with a good attitude towards preconception care. This finding is supported by a study done in Iran [40]. This could be explained by the fact that when education levels rise, critical thinking abilities do as well. This allows women to use technology to acquire health information and have discussions with healthcare providers about their health, which in turn changes their behavior [41].

This study showed that modern family planning use history was significantly associated with a good attitude towards preconception care. This finding is matched with studies done in Nigeria [24], Malaysia [49], and Kelantan [31]. This due to the fact that women who actively engage in family planning are likely to have a proactive approach to their reproductive health and it is a valuable source of information regarding preconception care [50].

Moreover, this study showed that pre-conception counseling was significantly associated with a good attitude towards preconception care. This finding is supported by a study done in Egypt [42]. This due to the fact that women who are informed about preconception care realize and appreciate its importance, and as a result, they may adopt a favorable attitude toward preconception care [51].

Furthermore, this study revealed that knowledge of preconception care was significantly associated with a good attitude towards preconception care. This is in line with a study done in Egypt [42]. This is due to the fact that women who are aware of preconception care and what to do before getting pregnant are more likely to act in accordance with their knowledge and exhibit risk-reduction behaviors [52].

### Limitation of the study

This study only includes women of reproductive age with chronic disease who had follow-up at chronic medical follow-up OPD only; it did not include other women who have follow-up at other OPDs.

There was the possibility of recall and social desirability bias.

## Conclusion

This study indicated that women’s knowledge and attitude towards preconception care among women of reproductive age with chronic disease were found to be low. Factors associated with good knowledge of preconception care were level of education, primiparity, preconception counseling, and longer duration of disease (lasting five years and above). This study also noted that older age, level of education, history of modern family planning, preconception counseling, and knowledge of preconception care were significant predictors of women’s attitude towards preconception care.

This highlights the need for targeted interventions to enhance women’s knowledge and attitude towards preconception care. Important measures include promoting secondary education and carrying out educational campaigns in collaboration with the health and education sectors. It is critical to carry out awareness campaign, and incorporating preconception counseling into routine medical follow-up care. Additionally, encouraging the use of modern family planning methods, and ensuring access to family planning services. Furthermore, it is crucial to provide tailored support and counseling by considering level of education, disease duration, parity, and the stages of their reproductive years.

## Data Availability

Full data for this research is available through the corresponding author upon reasonable request.

## Abbreviations

ANC: Antenatal Care
LMICs: low and middle income countries
OPD: Outpatient Department
PCC: Preconception Care

## References

[1] World Health Organization. Meeting to develop a global consensus on preconception care to reduce maternal and childhood mortality and morbidity: World Health Organization Headquarters, Geneva, 6–7 February 2012: meeting report. 2012.

[2] Tydén T. Why is preconception health and care important? Taylor & Francis; 2016. p. 207. 10.1080/03009734.2016.1211776 PMC5098481 27487464

[3] UNICEF, UNFPA. Trends in maternal mortality 2000 to 2017: estimates by WHO. World Bank Group and the United Nations Population Division; 2019.

[4] Ethiopia ICF, Central Statistical Agency - CSA/ (2017). Ethiopia Demographic and Health Survey 2016.

[5] Mason E, Chandra-Mouli V, Baltag V, Christiansen C, Lassi ZS, Bhutta ZA (2014). Preconception care: advancing from ˜important to do and can be done to ˜is being done and is making a difference. Reproductive Health.

[6] Ethiopian Fact Sheet. Maternal and Child Health–. 2020.

[7] Amugsi DA, Dimbuene ZT, Mberu B, Muthuri S, Ezeh AC (2017). Prevalence and time trends in overweight and obesity among urban women: an analysis of demographic and health surveys data from 24 African countries, 1991–2014. BMJ open.

[8] Federal Democratic Republic of Ethiopia MoH. National strategic plan for the prevention and control of major non-communicable diseases, 2013–2017 efy (2020/21-2024/25). July 2020.

[9] Tesfay FH, Zorbas C, Alston L, Backholer K, Bowe SJ, Bennett CM. Prevalence of chronic non-communicable diseases in Ethiopia: a systematic review and meta-analysis of evidence. Front Public Health. 2022;10. 10.3389/fpubh.2022.936482 PMC9385028 35991039

[10] Bearak J, Popinchalk A, Alkema L, Sedgh G (2018). Global, regional, and subregional trends in unintended pregnancy and its outcomes from 1990 to 2014: estimates from a bayesian hierarchical model. Lancet Global Health.

[11] Steel A, Lucke J, Adams J (2015). The prevalence and nature of the use of preconception services by women with chronic health conditions: an integrative review. BMC Womens Health.

[12] Dean SV, Imam AM, Lassi ZS, Bhutta ZA (2013). Systematic review of preconception risks and interventions.

[13] Dewau R, Muche A, Fentaw Z, Yalew M, Bitew G, Amsalu ET (2021). Time to initiation of antenatal care and its predictors among pregnant women in Ethiopia: Cox-gamma shared frailty model. PLoS ONE.

[14] Bortolus R, Oprandi NC, Morassutti FR, Marchetto L, Filippini F, Tozzi AE (2017). Why women do not ask for information on preconception health? A qualitative study. BMC Pregnancy Childbirth.

[15] Corton MM, Leveno KJ, Bloom SL, Hoffman BL. Williams Obstetrics 24/E (EBOOK). McGraw Hill Professional; 2014.

[16] Setegn M (2021). What women do before pregnancy A preconception care of women in Mizan Aman town Southwest Ethiopia a mixed study. Prim Health Care: Open Access.

[17] Oketch DA, Onguru D, Ogolla S, Geoffrey A. Factors Influencing Preconception Care Services Among Women of Reproductive Age at Jaramogi Oginga Odinga Teaching and Referral Hospital, Kisumu. 2021.

[18] Sabr Y, Al-Zahrani NF, Labani RM, Alrasheed RA. Knowledge, attitudes and practice of preconception care among women attending a university tertiary hospital in Riyadh: Cross sectional study. 2021.

[19] Temel S, Erdem Ö, Voorham TA, Bonsel GJ, Steegers EA, Denktaş S (2015). Knowledge on preconceptional folic acid supplementation and intention to seek for preconception care among men and women in an urban city: a population-based cross-sectional study. BMC Pregnancy Childbirth.

[20] Sutan R, Mohamed N, Tamil AM, Yusof A (2016). A case control study on maternal health-seeking behavior at pre-pregnancy stage among mothers with low birth weight babies. Womens Health Gynecol.

[21] Landon MB, Galan HL, Jauniaux ER, Driscoll DA, Berghella V, Grobman WA (2020). Obstetrics: normal and problem pregnancies.

[22] Adugna A. Amhara Demography and Health survey. 2018.

[23] Cochran WG. Sampling techniques. Wiley; 1977.

[24] Adeyemo AA, Bello OO (2021). Preconception care: what women know, think and do. Afr J Med Health Sci.

[25] Fikadu K, Wasihun B, Yimer O (2022). Knowledge of pre-conception health and planned pregnancy among married women in Jinka town, southern Ethiopia and factors influencing knowledge. PLoS ONE.

[26] Akinniyi AA, Olamide OO (2017). Missed medical appointment among hypertensive and diabetic outpatients in a tertiary healthcare facility in Ibadan, Nigeria. Trop J Pharm Res.

[27] CDC. Arthritis Comorbidities 2019,May 17 [cited 2022 August 1]. Available from: https://www.cdc.gov/arthritis/data_statistics/comorbidities.htm.

[28] Boakye-Yiadom A, Sagru-Larr E, Oduro E, Asumadu Okd S, Ja (2020). Asare Ro. Preconception care: awareness, knowledge, attitude and practice of pregnant women, tamale west hospital. Am J Health Med Nurs Pract.

[29] Ayalew Y, Mulat A, Dile M, Simegn A (2017). Women`s knowledge and associated factors in preconception care in adet, west gojjam, northwest Ethiopia: a community based cross sectional study. Reproductive Health.

[30] Jusoh N, Ismail TAT, Hamid NAA (2020). Knowledge of pre-pregnancy care among women with high risk pregnancy in perak, Malaysia: what are the factors?. Malaysian J Public Health Med.

[31] Kasim R, Draman N, Kadir AA, Muhamad R. Knowledge, attitudes and practice of preconception care among women attending maternal health clinic in Kelantan. Educ Med J. 2016;8(4).

[32] Fowler JR, Mahdy H, Jack BW. Preconception counseling. 2017.

[33] Kassa A, Yohannes Z (2018). Women`s knowledge and associated factors on preconception care at Public Health Institution in Hawassa City, South Ethiopia. BMC Res Notes.

[34] Fekene DB, Woldeyes BS, Erena MM, Demisse GA (2018). Knowledge, uptake of preconception care and associated factors among reproductive age group women in West Shewa Zone, Ethiopia, 2018. BMC Womens Health.

[35] Teshome F, Kebede Y, Abamecha F, Birhanu Z (2020). What do women know before getting pregnant? Knowledge of preconception care and associated factors among pregnant women in Mana district, Southwest Ethiopia: a community-based cross-sectional study. BMJ open.

[36] Madanat AY, Sheshah EA (2016). Preconception care in Saudi women with diabetes mellitus. J Fam Commun Med.

[37] Nepali G, Sapkota SD (2017). Knowledge and practice regarding preconception care among antenatal mothers. Int J Perceptions Public Health.

[38] Gamshe E, Demissie D. Perinatal factors affecting knowledge and utilization of Preconception Care among pregnant women at selected hospitals in Addis Ababa: a cross-sectional study. MJH; 2022.

[39] Olowokere A, Komolafe A, Owofadeju C (2016). Awareness, knowledge and uptake of preconception care among women in Ife Central Local Government Area of Osun State, Nigeria. J Community Med Prim Health Care.

[40] Jafari F, Rashidi S (2017). Iranian women’s knowledge and attitude regarding preconception health: 12 years after integration into the primary health care network. J Nurs Midwifery Sci.

[41] Zimmerman E, Woolf SH. Understanding the relationship between education and health. NAM Perspect. 2014.

[42] Emam E, Abd El Rheem A, Ghanem NHH, Hassan HE (2019). Knowledge and attitude of women and nurses regarding Pre-conception Care: a comparative study. Am Res Journals.

[43] Priani IF, Afiyanti Y, Kurniawati W (2019). Preparing pregnancy through preconception education training. Enfermeria Clin.

[44] Patel PG, Shah TA (2019). Assessment of knowledge and attitude regarding Pre-conceptional Care among newly married women residing at Urban areas of Vadodara City, Gujarat, India. Natl J.

[45] Moradi M, Fazeli N, khadivzadeh T, Esmaily H (2016). Application of Health Belief Model to assess knowledge and attitude of women regarding Preconception Care. J Midwifery Reproductive Health.

[46] Firouzi M, Ebrahimi A (2017). Knowledge and attitudes of women about preconception care. Qom Univ Med Sci J.

[47] Dlamini BB, Nhlengetfwa MN, Zwane I. Knowledge, Attitudes and Practices Towards Preconception Care Among Child Bearing Women. 2019.

[48] Ahmed K, Saeed A, Alawad A (2015). Knowledge, attitude and practice of preconception care among Sudanese women in reproductive age about rheumatic heart disease. Int J Public Health.

[49] Ehsan S, Mukhali H, Nik Mahdi NNR, Abd Aziz A, Jalaluddin A, Embong K (2022). Do our Diabetic patients Ready for safe pregnancy? Attitude towards Preconception Care and its Associated factors among women with diabetes attending Government Health clinics in Terengganu. Malaysian J Med Health Sci.

[50] Khan R, Shehata H. Preconceptional counseling. Preconceptional Med. 2012:19.

[51] Hou S-I (2014). Health education: theoretical concepts, effective strategies and core competencies. Health Promot Pract.

[52] Yahya R, Muhamad R, Yusoff HM (2012). Association between knowledge, attitude and practice on cardiovascular disease among women in Kelantan, Malaysia. Int J Collaborative Res Intern Med Public Health.

